# Mitochondrial Dynamics, ROS, and Cell Signaling: A Blended Overview

**DOI:** 10.3390/life11040332

**Published:** 2021-04-10

**Authors:** Valentina Brillo, Leonardo Chieregato, Luigi Leanza, Silvia Muccioli, Roberto Costa

**Affiliations:** Department of Biology, University of Padova, 35121 Padova, Italy; valentina.brillo@studenti.unipd.it (V.B.); leonardo.chieregato@studenti.unipd.it (L.C.); silvia.muccioli@studenti.unipd.it (S.M.); roberto.costa@unipd.it (R.C.)

**Keywords:** mitochondrial dynamic, cell signaling, ROS, cancer

## Abstract

Mitochondria are key intracellular organelles involved not only in the metabolic state of the cell, but also in several cellular functions, such as proliferation, Calcium signaling, and lipid trafficking. Indeed, these organelles are characterized by continuous events of fission and fusion which contribute to the dynamic plasticity of their network, also strongly influenced by mitochondrial contacts with other subcellular organelles. Nevertheless, mitochondria release a major amount of reactive oxygen species (ROS) inside eukaryotic cells, which are reported to mediate a plethora of both physiological and pathological cellular functions, such as growth and proliferation, regulation of autophagy, apoptosis, and metastasis. Therefore, targeting mitochondrial ROS could be a promising strategy to overcome and hinder the development of diseases such as cancer, where malignant cells, possessing a higher amount of ROS with respect to healthy ones, could be specifically targeted by therapeutic treatments. In this review, we collected the ultimate findings on the blended interplay among mitochondrial shaping, mitochondrial ROS, and several signaling pathways, in order to contribute to the dissection of intracellular molecular mechanisms involved in the pathophysiology of eukaryotic cells, possibly improving future therapeutic approaches.

## 1. Introduction

Mitochondria are intracellular organelles present in eukaryotic cells that evolutionarily originated from symbiotic resident proteobacteria [1]. These organelles are involved in many cellular functions, such as oxidative phosphorylation, the regulation of cell proliferation, differentiation, and death. Their different roles in several cellular processes are largely dependent on ATP and reactive oxygen species (ROS) production, both generated during oxidative phosphorylation [2]. Indeed, targeting mitochondrial metabolism with molecules able to specifically disrupt mitochondrial fitness and trigger cell death has become a promising strategy against several diseases [3].

Importantly, mitochondria are physically interconnected with other subcellular organelles, such as endoplasmic reticulum (ER), lipid droplets, Golgi apparatus, lysosomes, melanosomes, and peroxisomes [4]. Indeed, mitochondria–organelle contact sites represent real signaling hubs that are involved in multiple cellular functions, such as lipid trafficking, mitochondrial dynamics, calcium (Ca^2+^) flow, and ER stress, such that the contacts not only result in physical but also functional links that finely tune multiple signaling pathways.

Moreover, the capability to establish these interactions with other intracellular organelles is strongly dependent on mitochondria’s high attitude to fuse and divide, leading to modification of the intracellular mitochondrial network [5].

In addition, ROS figure as byproducts of oxygen consumption and cellular metabolism, and 45% of their total amount is related to mitochondria, specifically to Complex I and Complex III leakage of electrons, which is involved in superoxide (O_2_^−^) and hydrogen peroxide (H_2_O_2_) production [6]. While ROS production was at first believed to be only detrimental for the cell in physiological conditions, in the last two decades, it has been considered that its presence in a sublethal concentration could act as a secondary messenger that specifically modulates distinct cellular pathways [7] and mitochondrial dynamics and morphology [8]. As a consequence, ROS homeostasis is strictly regulated by enzymatic and nonenzymatic mechanisms, with the aim of maintaining balance among ROS production and scavenging [9]. Once this critical equilibrium is impaired, ROS overload is one of the main players in the onset of a plethora of different diseases, including cancer [6], where it exerts a dual regulation, influencing cell survival and oxidative stress, leading to cell death, as well as mediating redox signaling events beneficial for the progression of the disease [9].

In this review, we describe the known signaling pathways mediated by mitochondrial structure rearrangements or by mitochondrial ROS release, focusing also on possible therapeutic targets against disease formation.

## 2. Mitochondrial Dynamics: A Multiplayer Regulation

Proper mitochondrial integrity and physiology is essential for cell homeostasis. Mitochondrial fusion and fission dynamics, organelle transport, mitophagy, interaction with other organelles, such as the endoplasmic reticulum (ER) and the cytoskeleton, and genomic mitochondrial control are only a few of the several mechanisms involved in the fitness of these fundamental organelles. Thus, the improper surveillance on mitochondrial dynamics and partitioning on daughter cells can give rise to a wide spectrum of syndromes and diseases [5].

**Figure 1:**
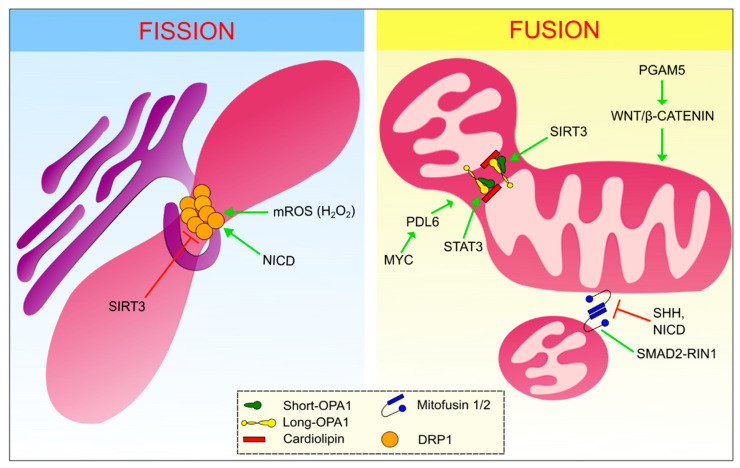
Modulation of fusion and fission processes. Left panel: the endoplasmic reticulum is wrapping the mitochondria in the site of fission, where polymers of dynamin-related protein 1 (DRP1) (main interactor in the fission process) are present. Right panel: the two different events of outer mitochondrial membrane (OMM) fusion and inner mitochondrial membrane (IMM) fusion are separately shown. Essentials components for OMM fusion are Mitofusins. In the IMM fusion process, instead, the role of long and short optic atrophy protein 1 (OPA1) is fundamental, as well as their interaction with cardiolipins. Green arrows point out the positive regulators of these processes, whereas the red ones represent the negative modulators.

Focusing on the fusion and fission processes, these are mainly regulated by proteins belonging to the dynamin-related family of large GTPases that utilize GTP hydrolysis to drive mechanical work on biological membranes (Figure 1) [10].

The membrane potential is also a crucial player in the mitochondrial fission process, by triggering dynamin-related protein 1 (DRP1) activity [11,12]. DRP1 translocates from the cytoplasm to the outer mitochondrial membrane (OMM) via a physical interaction with several adapter proteins, such as mitochondrial fission factor (MFF), mitochondrial dynamics protein 49 and 51 (MID49, MID51), and mitochondrial fission 1 protein (Fis1) [13]. Specifically, DRP1 firstly binds GTP through its GTP-binding site. This mediates a conformational change that allows DRP1 to interact with the OMM receptors and polymerize, encircling the mitochondria in a spiral fashion [14]. Once a complete turn is performed, contacts between the GTPase domains of the DRP1 polymers trigger the GTP hydrolysis causing the detachment of filaments from the OMM receptors and the constriction of the spiral [15]. Nevertheless, it seems that DRP1 itself is not sufficient to completely perform the fission process. In fact, it has been shown that Dynamin-2 is a fundamental component that works in concert with DRP1 in order to orchestrate sequential constriction events that ultimately lead to division [16]. Some authors suggested that the fission process occurs in sites that have been previously wrapped by a smooth ER protrusion. These ER–mitochondria contact sites act as the fission starting point, so that DRP1 can cooperate with the actin-nucleating protein inverted formin 2 (INF2) causing the accumulation of actin in the site of fission. Actin filament accumulation can ultimately facilitate the formation of the initial constriction, supporting the subsequent DRP1 activity [13,17].

In particular, ER–mitochondria contact sites, also known as mitochondria–ER contacts (MERCs), are relevant for mitochondrial fitness and plasticity regulation. Therefore, the characterization of the proteins involved in MERCs revealed the presence of several players that allow the connection between the two organelles. In fact, the existence of dynamic bridges that consist of proteins inserted in the OMM, such as voltage-dependent anion channel (VDAC), physically connected to the ER membrane proteins (such as inositol 1,4,5-triphosphate receptor (IP3R)), by linker proteins (e.g., glucose-regulated protein (GRP75) and transglutaminase type 2 (TG2)), contribute to the modulation of many mitochondrial events such as lipid trafficking, Ca^2+^ homeostasis, and ER stress [16]. Moreover, the functional role of such contacts is highlighted by their involvement in several pathologies, such as diabetes, neurodegenerative diseases, and cancer [18]. Interestingly, very recently, it was demonstrated that downregulation of transglutaminase type 2, which is involved in ER–mitochondria contacts [19], is linked to a decrease in canonical Wnt signaling targets, such as β-catenin and Lymphoid enhancer binding factor 1 (LEF1), suggesting new possible ways of modulating Wnt-dependent proliferation, strongly associated with diseases development [20].

Since mitochondria are characterized by a double membrane, the fusion process requires the coordination of two separate events which occur almost simultaneously. Indeed, mitochondrial fusion is a mechanism mediated by MFN1 and MFN2 at the level of the OMM and by optic atrophy protein 1 (OPA1) in the inner mitochondrial membrane (IMM) [10,17]. While MFN1 and MFN2 must both be present in the OMM in order to mediate outer membrane fusion, it is enough for OPA1 to be present in only one of the IMMs to mediate inner membrane fusion [10]. Fusion onset is established by the docking of two Mitofusin molecules. This interaction mediates conformational changes that trigger Mitofusin-mediated GTP hydrolysis and the subsequent OMM fusion [17]. 

Concerning IMM fusion, the *opa1* gene encodes eight different long isoforms [21], and all of them are firstly inserted into the IMM thanks to the presence of the mitochondrial targeting sequence (MTS), which is subsequently cleaved by the matrix processing protease (MPP). Once inserted into the IMM, OPA1 isoforms can be processed by two inner membrane peptidases: zinc metallopeptidase (OMA1) and ATP-dependent zinc metalloprotease (YME1L1), thus forming the essential short form of OPA1. Up to now, it seems that both the isoforms are required in order to guarantee the correct and physiological levels of mitochondrial fusion [22]. Then, the heterodimer of one IMM, formed by long-OPA1 and short-OPA1, interacts with cardiolipin of the other IMM and mediates IMM fusion [17]. It is important also to remember that OPA1 is essential in cristae structure maintenance [10].

## 3. Mitochondrial Plasticity and Cell Signaling: A Two-Way Interaction

In recent years, numerous studies pointed out links between key oncogenic signaling pathways and mitochondria [13]. These networks among the two players have been deeply explored, revealing not only that mitochondrial plasticity could be influenced by distinct cellular pathways, but also that mitochondrial shaping could be crucial for the modulation of several signaling cascades.

Indeed, Malhotra et al. explored the role of Sonic Hedgehog (Shh) signaling in mitochondrial biogenesis regulation in cerebellar granule neuron precursors (CGNPs), the progenitor of Shh-associated medulloblastoma. Surprisingly, the authors observed a decreased mitochondrial membrane potential (MMP) and overall ATP production in CGNP cells upon Shh induction, along with an increase in glycolysis levels, which resulted in higher intracellular acidity leading to mitochondrial fragmentation and reduced cristae network formation. These results are quite controversial since decreased MMP is usually linked to a reduction in proliferation and apoptotic induction. Inversely, these cells are characterized by a rise in the proliferation rate and absence of cell differentiation. This effect seems to be derived from the Shh-mediated lowering of MFN1 and MFN2 activity. Really interesting is that the phenotype can be rescued by the inhibition of the Shh pathway, as well as through the downregulation of DRP1, remarking the importance of the delicate balance between fission and fusion mechanisms involved in mitochondrial biogenesis [23].

Canonical Wnt-signaling is involved in a plethora of different cellular functions, such as neuroprotection, stemness maintenance, self-renewal, and regulation of mitochondrial dynamic inside eukaryotic cells [24,25,26]. Strikingly, recent studies demonstrated a novel mechanism via which damaged mitochondria promote restoration of the mitochondrial pool through the activation of canonical Wnt-signaling via the Pgam5/β-catenin axis. Presenilins-associated rhomboid-like (PARL)-mediated cleavage of the mitochondrial Serine/threonine-protein phosphatase Pgam5 occurs in stressed mitochondria characterized by a decreased membrane potential. This cleaved and cytosolic form seems to be able to interact with Axin at the level of the β-catenin destruction complex. Such interaction counters the casein kinase 1 α (CK1α)- and glycogen synthase 3 (GSK3)-mediated phosphorylation of β-catenin, thus avoiding its degradation and leading to an increased transcriptional activity performed through the activation of the canonical Wnt/β-catenin axis. This cell-intrinsic stimulation of the Wnt/β-catenin cascade can trigger the mitophagy process, in order to degrade or recycle the old and dysfunctional mitochondria, thus supporting the process of mitochondrial biogenesis [27].

Several other pathways are involved in the regulation of mitochondrial dynamics. For instance, it has been shown that the activity of mothers against decapentaplegic homolog (Smad) proteins—mediators of the transforming growth factor β (TGF-β) signaling—are able to modulate mitochondrial fusion when present in their inactive and cytoplasmatic form. In fact, SMAD2 can promote mitochondrial fusion through its interaction with MFN2 at the level of the OMM. In particular, a model has been established in which SMAD2 colocalizes with Ras and Rab interactor 1 (RIN1) and MFN2 at the level of the OMM and stimulates the process of fusion. Problems or mutations at the level of any of these characters can interfere with the modulation of mitochondrial dynamics, thereby favoring the development of different kinds of diseases, such as carcinogenesis and metabolic issues [28].

Indeed, increased mitochondrial fission is often associated with tumor formation, e.g., lung cancers and breast cancers. In particular, the presence of a mitochondrial fission and Notch signaling positive feedback loop has been elucidated in triple-negative breast cancers (TNBCs). In fact, it seems that mitochondrial fragmentation is linked to an increased cytoplasmic Ca^2+^ level, causing the subsequent activation of calcineurin. Therefore, calcineurin activates Notch signaling, increasing the level of the cleaved and active Notch intracellular domain (NICD) inside the nucleus. In turn, Notch signaling promotes the upregulation of DRP1 and the downregulation of MFN1, thus establishing a vicious cycle. Moreover, this positive feedback loop enhances the apoptotic resistance and survival of tumor cells through the Notch-mediated upregulation of the inhibitor of apoptosis (IAP) protein Survivin [29].

Moreover, some types of human breast cancers are characterized by dysregulated Myc signaling. Overexpression of Myc leads, among all the other targets, to the overexpression of Phospholipase D Family Member 6 (PLD6)—a phospholipase of the OMM involved in the regulation of lipid metabolism, which is able to mediate mitochondrial fusion in order to improve lipid metabolism, but which also cooperates with the increased nucleotide demand during DNA synthesis. This Myc-mediated metabolic reprograming, in part caused by the overstimulated mitochondrial fusion derived by PLD6 activity, strains cellular energy resources and leads to 5’ adenosine monophosphate-activated protein kinase (AMPK) activation. AMPK is also able to phosphorylate and inhibit yes-associated protein (YAP), and YAP inactivation is characteristic of some types of Myc-dependent triple-negative mammary carcinomas. Another effect mediated by PLD6-dependent mitochondrial fusion is also the increase in the levels of glutaminolysis, an essential process for tumor survival since MYC-driven cell growth depends on glutamine [30].

Additionally, mitochondrial fission is also correlated to other diseases, such as unilateral unilateral obstruction (UUO)-induced renal tubulointerstitial fibrosis. Indeed, it has been shown that Honokiol (2-(4-hydroxy-3-prop-2-enyl-phenyl)-4-prop-2-enyl-phenol, HKL) is able to stimulate the activity of Sirtuin 3 (SIRT3), which sequentially mediates the activation of OPA1 and decreases DRP1 expression, restoring the correct mitochondrial fusion and fission dynamics and normal mitochondrial shape and function. Thus, targeting mitochondrial dynamics can be a novel therapeutic approach for the treatment of acute or chronic kidney diseases [31].

Recently, the importance of signal transducer and activator of transcription 3 (STAT3) was also elucidated in the regulation of mitochondrial dynamics. Indeed, Zhang et al. demonstrated in diabetic mice and in albumin-treated proximal tubular HK-2 cells how anomalies resulting from diabetes, e.g., hyperglycemia and ROS, can mediate the overexpression or overactivation of the dipeptidyl peptidase-4 (DPP4) enzyme, leading to DPP4-mediated cleavage of stromal cell-derived factor-1α (SDF-1α) and suppression of the SDF-1α/C-X-C Motif Chemokine Receptor 4 (CXCR4) phosphorylation of STAT3 at the level of serine-727. Thus, this impedes STAT3 translocation into the mitochondria and its interaction with OPA1, ultimately leading to increased mitochondrial fragmentation. This result highlights novel targets for managing diabetic kidney disease [32].

The connection between STAT3 and mitochondrial fusion protein OPA1 has also been described in a myocardial ischemia and reperfusion mouse model. In this study, it was demonstrated how κ-opioid receptor (κ-OR) activation mediates mitochondrial fusion through enhanced OPA1 expression. In particular, this suggests that κ-OR activation can stimulate STAT3 phosphorylation at the level of tyrosine-705, allowing its nuclear translocation where it can mediate OPA1 overexpression. This result allows novel insight into therapeutic strategies for myocardial ischemia and reperfusion injury [33].

Thus, STAT3 induction of mitochondrial fusion through the modulation of OPA1 seems to be quite clear. Nevertheless, a more in-depth investigation is still needed into the effective mechanism.

## 4. Mitochondrial ROS in the Modulation of Cell Signaling

ROS are small molecules that figure as byproducts of oxygen consumption and cellular metabolism, which derive from the partial reduction of molecular oxygen. The most known molecules among the ROS family are the highly unstable oxygen free radicals, superoxide (O_2_^−^) and hydroxyl (OH^−^), which can be converted into more stable non-radical and diffusible forms, e.g., hydrogen peroxide (H_2_O_2_) or hypochlorous acid [6,34].

As it is well known, mitochondria represent one of the major contributors to ROS generation. In fact, it was recently demonstrated that, in resting C2C12 myoblasts, mitochondria account for the 45% of the total amount of reactive oxygen species produced inside the cell [35], and that up to 1% of the mitochondrial oxygen is utilized for superoxide production [36]. In addition, 11 distinct sites associated with substrate oxidation in the electron transport chain (ETC) in mammalian mitochondria resulted in the release of electrons involved in the production of superoxide (O_2_^−^) and hydrogen peroxide (H_2_O_2_). In particular, Complex I and Complex III are the main sources of ROS both in healthy and in pathological conditions, which are required for a plethora of biological processes such as cell differentiation and proliferation, oxygen cell sensing, and Hypoxia-inducible factor (HIF) activation [7,37,38,39].

Inside the mitochondria, mitochondrial ROS are mainly produced by Nicotinamide adenine dinucleotide phosphate (NADPH) oxidases (NOX) and, to a minor extent, by other enzymes such as cyclooxygenases (COX), lipoxygenases, xanthine oxidases, and cytochrome P450 enzymes [34,40,41,42,43]. Moreover, the electron transport chain is intrinsically leaky; indeed, even in physiological conditions, 0.2–2% of the electrons generated by the respiratory chain are not coupled with the production of ATP but contribute to the generation of superoxide anion (O_2_^−^) or hydrogen peroxide (H_2_O_2_) due to their premature interaction with oxygen. Thus, a minor percentage of ROS is physiologically released during all the respiratory processes, playing a crucial role in mitochondria and cell fate [44].

Actually, ROS generation is involved in the regulation and induction of both physiological and pathological cellular pathways. For a long time they were considered with a negative connotation in physiological conditions, being responsible for the induction of oxidative stress and consequent apoptosis and necrosis, ultimately resulting in alterations in cell survival rate [45]. Indeed, mitochondrial dysfunction in the ETC is strongly linked to an unregulated release of mitochondrial ROS, which causes both DNA and macromolecular oxidative damage, leading to the development of degenerative pathologies and biological aging [46]. For this reason, ROS homeostasis must be strictly regulated by enzymatic and non-enzymatic mechanisms, such as superoxide dismutases (SODs), catalases, glutathione peroxidases (GPX), peroxiredoxins (PRX), thioredoxins (TRX), and vitamins A, C, and E (for extended reviews, see [9,47,48,49]).

Accordingly, ROS are well recognized mediators of DNA damage, affecting the DNA damage response (DDR), and their accumulation can also induce mitochondrial DNA lesions, strand breaks, and degradation of mitochondrial DNA [50]. Specifically, ROS-induced DNA damage and the consequent inability to evoke the DNA repair system are responsible for Cellular tumor antigen p53 (p53) activation and mitochondrial-mediated apoptosis, a pathway that is elicited by different anticancer drugs, leading to the suggestion that ROS modulators could be promising for cancer targeting [51,52].

Interestingly, in the latest two decades, a new role for mitochondrial ROS emerged. In fact, sublethal concentrations of ROS could act as potential secondary messengers which could be used to specifically modulate distinct cellular pathways, introducing new possible therapeutic approaches [7,53]. Specifically, via the reversible oxidation of specific cysteine (and also methionine) residues within redox-sensitive proteins, ROS can modify a putative target protein activity or conformation, altering the signal transduction. In this regard, ROS can act on phosphatases, kinases, proteases, and transcription factors [54], regulating growth factor cascades, cell proliferation and differentiation, cellular oxygen sensing, and hypoxia (and the consequent angiogenic stimulation), while also finely tuning aging-related mechanisms, immunity responses, inflammation, and autophagy [9,44,45,53,55,56,57].

Moreover, a role for ROS in the modulation of mitochondrial dynamics was recently elucidated, suggesting a link between the redox homeostasis of the cell and the regulation of mitochondrial morphology [8]. In particular, high levels of ROS, if not counterbalanced by an efficient antioxidant system, promote mitochondrial fragmentation, swelling, or shortening, whereas a reduction in ROS leads to mitochondrial filamentation. In fact, exogenous concentrations of hydrogen peroxide induced dose-dependent mitochondrial fragmentation in human umbilical vein endothelial cells (HUVECs) and the expression of several fusion and fission-related genes [58]; in C2C12 myocytes, hydrogen peroxide induced mitochondrial membrane depolarization, stimulating fragmentation involving an increased DRP1 activity [59,60]. On the contrary, lowering ROS levels in fibroblasts triggered MFN2-dependent mitochondrial filamentation [61]. The redox regulation of fission and fusion proteins by ROS is mediated by post-translational modifications, such as phosphorylation, ubiquitination, and sumoylation, in addition to the *S*-glutathionylation and *S*-nitrosylation of their Cys residues [62]. Moreover, ROS also act at the transcriptional level, stimulating the expression of factors that are involved in both redox regulation and mitochondrial dynamics; an example is the peroxisome proliferator-activated receptor gamma coactivator (PGC1α/β), which is redox-sensitive and associated with MFN2 regulation [63]. Another important role in the link among mitochondrial dynamics and ROS is played by AMPK; once activated, it phosphorylates MFF and DRP1, necessary for mitochondrial fission [62].

## 5. Mitochondrial ROS Involvement in Cancer

Interestingly, when an imbalance among the production and the scavenging of ROS species occurs, impaired ROS homeostasis results in the onset and the progression of various pathologies, including neurodegenerative diseases [64], diabetes [65,66], cardiovascular diseases [67], and cancer [6,68]. More specifically, it is clear that mitochondrial ROS can act in a dual mode during the progression of these pathologies; as oxidants, at elevated concentrations, they influence cell survival and oxidative stress, ultimately leading to cell death, whereas, at lower concentrations, they act as signaling molecules which mediate redox signaling events beneficial for the progression of the disease [9,37].

In addition, cancer cells are characterized by increased ROS levels with respect to normal cells; this is due to their abnormal metabolism, which exploits normal cell machinery in a constitutive way in order to maximize cellular growth and proliferation, to enhance aerobic glycolysis (the so-called “Warburg effect”) [9], and to promote altered expression of pro-tumorigenic networks (as for example, Kras and Myc overexpression [69,70]), as well as the inhibition of tumor suppressors [71]. Moreover, the accumulation of mutations in mitochondrial DNA (mtDNA), increased tumor-derived hypoxia, and mitochondrial shape changes, along with alterations in the antioxidant system and in cellular signaling pathways, all contribute to the increased ROS level in neoplastic cells [45].

High ROS levels have been demonstrated to be causative of a cascade of multiple events in cancer—perpetuating the tumorigenic transformation—including DNA damage [50], genetic instability, enhanced cell proliferation, cellular injury, cell death, and resistance to drugs [34]. Moreover, ROS species work as signaling intermediates in several pathways that are physiologically used by healthy cells in order to sustain both proliferation and cellular growth [71]. Crucial pathways that are activated by ROS accumulation are the mitogen activated-protein kinase (MAPK)/extracellular-regulated kinase 1/2 (ERK1/2) and phosphatidyl inositol 3 kinase (PI3K) signaling cascades that are mainly responsible for cell proliferation, growth, and survival. Indeed, ROS have been found to be involved in the inhibition of the phosphatase and tensin homolog (PTEN) via cysteine oxidation, thereby promoting Akt activity and positively regulating the PI3K pathway, which, in turn, results in higher proliferation rates [72].

**Figure 2:**
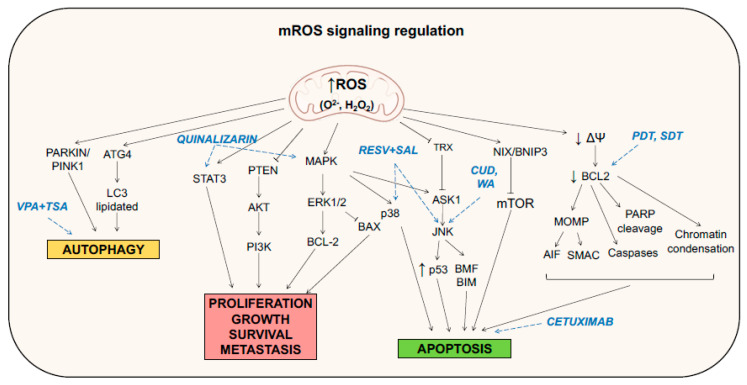
Mitochondrial reactive oxygen species (ROS) regulation of cellular signaling pathways. Many convergent signaling pathways that contribute to autophagy, proliferation, metastasis, and apoptosis are deeply modulated by an increase in mitochondrial ROS. In blue are depicted several drugs discussed in the text and reported in Table 1, which have been demonstrated to target key mediators of the pathways involved in ROS signaling.

Moreover, it was recently discovered that high concentrations of mitochondrial ROS in cancer stem cells (CSCs) promote cancer metastasis, via fatty acid β-oxidation, involving the activation of PI3K/AKT and ERK signaling, leading to epithelial-to-mesenchymal transition (EMT) [73]. In addition, Wang et al. demonstrated that, in colorectal cancer, elevated cholesterol levels increased ROS production, which, in turn, activated the MAPK signaling pathway, stimulating tumor progression (Figure 2) [74].

Even STAT3, which is activated in a plethora of cancers and controls the expression of multiple genes involved in tumor initiation, progression, and chemoresistance, has been proven to be regulated by mitochondrial ROS production. Normally, STAT3, in its inactive form, is present as a monomer in the cytoplasm, whereas, once activated by Janus activated kinases (JAKs), proto-oncogene tyrosine-protein kinase Src (Src), and MAP kinases, on its tyrosine-705 (Y705) and serine-727 (S727), it dimerizes, migrates into the nucleus, and regulates the transcription of several proliferative and antiapoptotic genes such as cyclins, Bcl-2, Bcl-xl, and Survivin [75,76]. Moreover, a distinct pool of STAT3 resides in the mitochondria and is responsible for the control of ETC, modulation of ROS production, Ras transformation, cellular growth, and protection from ischemia/reperfusion injuries through the regulation of the mitochondrial permeability transition pore (MPTP) [77]. Very recently, Lee et al. elucidated a role in hepatoma cell invasiveness of ROS-induced STAT3 activation, which in turn promoted Nrf2 transcription and syntaxin 12 expression [78].

Dysregulated mitochondrial dynamics have been reported in various diseases including cancer, and they can contribute to development, progression, and chemoresistance of tumors. Recent studies demonstrated that higher levels of ROS induce a DRP1-mediated mitochondrial fission in metastatic cancer and tumor-initiating cells, increasing migration and chemoresistance [79]. As an example, hypoxia-induced ROS in ovarian cancer cells are responsible for an increase in mitochondrial fission rate through the activation of DRP1 and downregulation of MFN1, leading to cisplatin resistance [80].

**Figure 3:**
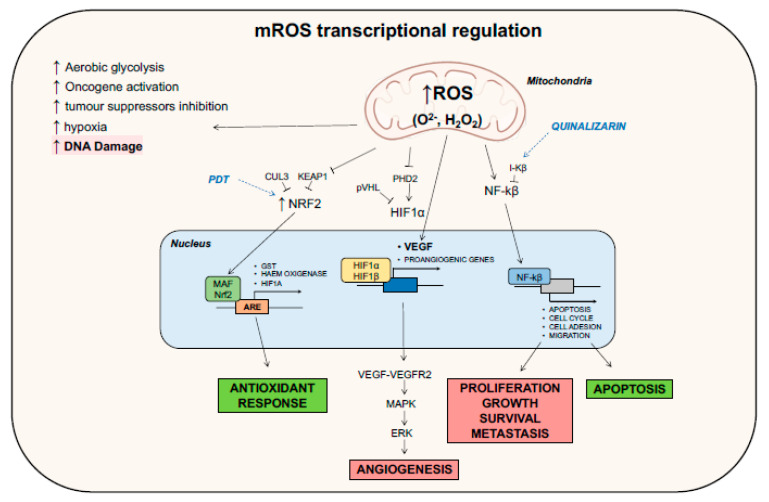
Mitochondrial ROS regulation of cellular processes at a transcriptional level. Antioxidant response, angiogenesis, proliferation, metastasis, and apoptosis are strictly regulated events by an increase in ROS production in the mitochondria (mROS). Indeed, mitochondrial ROS increase promotes the translocation into the nucleus of important factors that possess transcriptional activity, leading to the synthesis of genes related to these main events. In blue, several drugs that target intermediates of different signaling cascades are shown, as reported in Table 1.

Generally, high levels of ROS production are counterbalanced by enhanced levels of antioxidant and scavenging activity, carefully maintaining a redox balance in order to avoid reaching a toxic amount of ROS which would lead to programmed cell death by apoptosis or necrosis. The most important way in which tumor cells potentiate their antioxidant system is through the activation of the nuclear factor erythroid 2-related factor 2 (Nrf2) [81]. Normally, this protein interacts with Kelch-like Enoyl CoA hydratase (ECH)-associated protein 1 (KEAP1) and ubiquitine ligase cullin3 (CUL3) and is targeted for proteasomal degradation. In elevated ROS condition, the oxidation of several cysteine residues in KEAP1 releases Nrf2, which translocates into the nucleus, associates with the MAF proteins, and binds to antioxidant-responsive elements (AREs) within the regulatory regions of several antioxidant genes [71], including those encoding Glutathione (GSH) *S*-transferase (GST), heme oxygenase 1 [53], and HIF1α (Figure 3) [82].

Along with cell proliferation, other ROS-dependent signaling pathways are important for the adaptation of tumor cells to hypoxia-induced metabolic stress. Generally, in nonhypoxic conditions, hypoxia-inducible factors (HIFs) form heterodimers made up of two subunits: HIF1α and HIF1β. The oxygen-sensitive HIF1α is then hydroxylated by prolyl hydroxylases (PHDs) and targeted to proteasomal degradation due to its ubiquitylation by von Hippel–Lindau protein [83]. Instead, hypoxia stabilizes the HIFs, and the larger production of ROS inhibits PHD2 [84], thereby stabilizing HIF1α that, in turn, translocates into the nucleus, dimerizes with HIF1β, and regulates the expression of proangiogenic genes, including vascular endothelial growth factor (VEGF) [54]. Eventually, ROS are also able to directly enhance VEGF production at a transcriptional level. Finally, once bound to its receptor VEGFR2, VEGF promotes the proangiogenic signaling cascade, leading to activation of the ERK/MAPK pathway (Figure 3) [54,85].

An excessive level of ROS could give rise to apoptotic and autophagic responses, through the interaction with fundamental signaling molecules. Indeed, either extrinsic or intrinsic apoptosis has been demonstrated to be activated by mitochondrial ROS. For instance, ROS oxidation of thioredoxin (Trx) mediates the separation of Thx from Apoptosis signal-regulating kinase 1 (ASK1), a mitogen activated protein (MAP) kinase kinase kinase (MAPKKK) that upstream regulates c-Jun n-terminal kinase (JNK) pathways. ASK1 homo-oligomerization and activation by autophosphorylation phosphorylates JNK that, in turn, phosphorylates Bim and Bmf proteins, further activating Bcl-2-associated death promoter Bax and Bak, to initiate apoptosis. Moreover, JNK can increase p53 expression inducing apoptosis [68]. Additionally, other signaling cascades have been demonstrated to drive apoptosis through higher ROS levels, such as the mitogen-activated protein kinases (MAPKs), the signal transducer and activator of transcription-3 (STAT3), and the nuclear factor κB (NF-κB) signaling pathways. MAPK signaling includes extracellular-signal-regulated kinase (ERK), JNK, and p38, which regulate not only proliferation but also a variety of other cellular behaviors [86]. In fact, JNK (as previously reported) and p38 are considered mediators of apoptosis and are activated through phosphorylation by MAPK in response to several stress signals, including ROS. On the contrary, ERK, which is activated by growth factors, is considered pro-survival and oncogenic, and it antagonizes apoptosis by phosphorylating proapoptotic Bax and antiapoptotic Bcl-2 proteins, inhibiting and promoting their functions, respectively (Figure 2) [87]. Activated ROS production also plays a role in JAK2/STAT3 signaling suppression and subsequent apoptosis induction; for example, Cao et al. demonstrated that CYT997, a novel synthetic microtubule-disrupting agent, through the upregulation of mitochondrial ROS, triggers protective autophagy and inhibits the JAK2/STAT3 pathway, inducing gap 2 (G2)/mitosis (M) arrest and apoptosis in gastric cancer cells [88].

Furthermore, the activity of NF-κB, which is part of a family of signal-responsive transcription factors, has been shown to be modulated by ROS levels. In fact, in the classical pathway, NF-κB can be maintained inactive within the cytoplasm through interactions and binding to inhibitor of κB (i-κB) in normal cells, whereas it is constitutively activated in cancer cells; the phosphorylation of i-κB protein results in it being targeted by protease, releasing NF-κB that is translocated to the nucleus where it acts as transcription factor, leading to the expression of genes related to apoptosis, cell cycle control, adhesion, and migration [89]. All these processes are strictly related to tumor progression [90]. Chen et al. recently discovered that deferoxamine (DFO), an iron chelator and anticancer drug, was able to increase mitochondrial ROS and, in turn, elicit NF-κB and TGF-β pathways, promoting migration of a TNBC cell line MDA-MB-231 (Figure 2) [91].

Lastly, a complex interconnection among ROS and autophagy is present in cancer cells. Autophagy stands for the regulated self-degradative process in mammalian cells where unnecessary or dysfunctional cytoplasmic organelles are degraded in the lysosomes. This process has been demonstrated to be elicited by several anticancer drugs [92]. Autophagy driven by mitochondrial ROS possesses a double role; the first is to decrease the intracellular ROS level, mediating the mitophagy (degradation of damaged mitochondria) that contributes to oxidative stress. Mitophagy is achieved through the NIX/ B-Cell lymphoma 2 (BCL2) and adenovirus E1B 19kDa-interacting protein 3 like (BNIP3L) and ubiquitin-protein ligase PARKIN/PTEN induced putative kinase 1 (PINK1) molecular pathways [93,94]. On the other hand, elevated ROS levels contribute to defective autophagy in cancer cells, leading to autophagic cell death [95]. As an example, hydrogen peroxide, through the activation of BNIP3, inhibits mammalian target of rapamycin (mTOR) activity and induces autophagy in C6 glioma cells after sanguinarine treatment [96]. Moreover, under starvation conditions, autophagy related 4 (ATG4) protease becomes a target of mitochondrial produced hydrogen peroxide that oxidates its cysteine residue, mediating its inactivation, and promoting the lipidation of LC-3, starting the autophagosome formation process (Figure 2) [97] (for extended reviews on ROS control of autophagy, see [98,99]).

## 6. Hints for Anticancer Therapy: Exploitation of Mitochondrial ROS

As clearly stated in the previous paragraphs, tumor cells generate and maintain high levels of ROS to preserve pro-tumorigenic signaling cascades, granting proliferation, growth, and metabolic adaptation. However, their level must be tightly regulated by the antioxidant system of the cell, in order to not exceed the toxic threshold ROS level, preventing cell death due to oxidative stress. This duality represents the specific challenge in the effort to find an effective ROS therapy in cancer.

Indeed, manipulating ROS in the context of cancer treatment is a promising approach recently developed, either by decreasing or by increasing their levels in cancer cells. The first approach relies on trying to decrease ROS levels while increasing antioxidant systems, in order to diminish the pro-tumorigenic activity of ROS. The reduction in ROS levels not only decreases cell survival and proliferation but also reduces DNA damage and genetic instability, lowering the pro-tumorigenic signaling and the exacerbation of the tumorigenicity. A great variety of studies aimed at investigating the effects of a range of antioxidants on tumor growth and yielded different outcomes, from no effect to, in some cases, increased cancer-related mortality [109]. On the other hand, metformin, a pleiotropic drug that targets mitochondrial complex I with antineoplastic functions, seems to suppress ROS production, decreasing ROS levels and inhibiting inflammatory signaling and metastatic progression in breast cancer [110]; moreover, metformin decreased the viability of Mia PaCa and PANC1 pancreatic ductal adenocarcinoma cell lines through the reduction in intracellular ROS, increasing MnSOD and decreasing NOX2 and NOX4 [111].

The second approach consists of pushing the ROS concentration over the threshold of toxicity, selectively killing tumor cells by disabling antioxidants and activating different cell death processes such as apoptosis, necrosis, and autophagy-mediated cell death. Necrosis, for example, is a programmed cell death characterized by organelle swelling and membrane rupture. As apoptosis, it involves a controlled signaling cascade which requires the receptor-interacting protein kinase 1 (RIP1)/ receptor-interacting protein kinase 3 (RIP3) complex, whose formation was proven to be regulated by mitochondrial ROS [112]. A novel type of cell death is ferroptosis, an iron-dependent programmed cell death occurring when the intracellular levels of lipid reactive oxygen species exceed the activity of glutathione peroxidase 4 (GPX4), leading to the collapse of redox homeostasis. Mitochondria are focal hubs for iron metabolism and homeostasis; moreover, the free and redox active iron pool has been demonstrated to participate in the accumulation of mitochondrial ROS, which can interact with polyunsaturated fatty acids, leading to lipid peroxidation, initiating ferroptosis in cancer and healthy cells [113]. Lastly, pyroptosis could also be an option. This mechanism is mediated by the gasdermin family, accompanied by inflammatory and immune responses; in the last few years, it has been considered a potential cancer treatment strategy [114]. One of the latest updates in ROS-exploiting cancer therapy, in fact, identifies iron as an amplifier of ROS signaling to induce pyroptosis (a lytic programmed cell death initiated by inflammasomes), via the Tom20/Bax/caspase-3-cleaved gasdermin E (GSDME) pathway in melanoma cells [115].

Chemotherapy, the most common treatment in cancer, in the majority of cases, elevates intracellular levels of ROS, in general pushing the cancer cell over a threshold to induce cell death; this is one of the proposed mechanisms via which chemotherapeutics provoke tumor regression. There are two causes for the increase in ROS level in the tumor cell: mitochondrial ROS generation and inhibition of the antioxidant system [116]. Intracellular ROS increase promotes a series of signaling cascades, including the activation of MAPK and NF-κB pathways; moreover, DNA damage induced by ROS accumulation can promote p53 accumulation, activating the p53/Bax pathway and resulting in apoptosis [117].

The combinatorial therapy against breast cancer using resveratrol (RESV)—a natural polyphenol having antiproliferative activity against breast cancer cells—and salinomycin (SAL)—a monocarboxylic polyether ionophore—in MCF7 cell lines has been observed to elicit an apoptotic response through the enhancement of mitochondrial ROS, because of mitochondrial impairment. In fact, after the combinatorial treatment, ROS increase induced mitochondrial membrane potential disruption, decreasing the expression of Bcl-2. This led to the activation of caspases 7,8,9, chromatin condensation, and Poly adenosine diphosphate (ADP)-ribose polymerase (PARP) cleavage, inducing apoptosis. In addition, ROS activated the MAPK pathway, which responds to cellular stress and metabolism by phosphorylating JNK and p38 and leading to apoptosis [100]. In the same direction, Xia et al. studied for the first time on colorectal cancer cells the effect on tumor cells of Withaferin A (WA), an active steroidal lactone derived from *Withania somnifera* that exhibits antitumor activity in several cancers, including breast cancer, lung cancer, and pancreatic cancer, via ROS production. They validated the hypothesis that ROS production, driven by mitochondrial dysfunction, inhibited cell growth and increased apoptosis; the reduction in mitochondrial membrane potential started the traditional apoptotic cascade (decrease on Bcl-2/Bax ratio, subsequent activation of caspase 3–9) and activated of the JNK pathway [101]. Carnosic acid (CA), an antioxidant compound derived from *Rosmarinus officinalis*, was able to induce apoptosis in HCT116 colon cancer cell line via ROS generation and inactivation of STAT3 signaling. Specifically, treatment with CA, generating ROS, diminished the phosphorylation of STAT3, JAK2, and Src kinases (it is likely that ROS may cause oxidative modification of Cys residues on these proteins), decreasing also the expression of STAT3 gene products, such as D-cyclins and survivin [76]. Quinalizarin, an anthraquinone component isolated from Rubiaceae, has been demonstrated to link ROS generation to MAPK, STAT3, and mitochondrial dynamics and inheritance during cell division, as well as the development and disease NF-κB signaling pathways, leading the MCF7 breast cancer cell line and A549 lung cancer cell line to cell-cycle arrest and caspase-dependent apoptosis [102,103]. Cucurbitacin (CuD), a common phytochemical derived from *Trichosanthes kirilowii,* was used in Capan-1 pancreatic cancer cell line, demonstrating that the drug-induced ROS production induced G2/M cell-cycle arrest and mediated the p38/MAPK pathway, promoting cell death (Figure 2 and Figure 3) [104].

More studies, instead, are needed to understand the exact mechanism and correlation among mitochondrial ROS production and mitochondrial dynamics in cancer, to utilize these findings for therapeutic purposes, in order to overcome chemoresistance and/or to improve patient prognosis [79]. Meanwhile, Chuang et al. very recently demonstrated that imiquimod (IMQ), a Toll-like receptor (TLR) 7 ligand, induced severe ROS production that in turn caused mitochondrial membrane potential loss, mitochondrial fission, and mitophagy in skin cancer cells [105]. Moreover, isorhamnetin (IH), a flavonoid that is present in plants of the Polygonaceae family, in combination with chloroquine (CQ), was able to induce apoptosis in triple-negative breast cancer cells, via an ROS-mediated phosphorylation of CaMKII/Drp1, leading to Bax translocation and release of cytochrome c, mitochondrial fission, caspase activation, and apoptosis [106].

In the last few years, the role of ROS in cancer therapy, especially the increase in ROS levels elicited by targeted therapy, has received more and more attention; monoclonal antibodies and small-molecule inhibitors, which specifically target tyrosine kinases, have been demonstrated to show ROS-mediated anticancer effects, eliciting signaling cascades that provoke apoptosis [68]. Moreover, other targeted therapies such as proteasome inhibitors, histone deacetylase (HDAC) inhibitors (HDACi), and STAT3 inhibitors have been shown to sensitize tumor cells by increasing the level of ROS. Cetuximab, in combination with oridonin, inducing ROS production, enhanced mitochondrial apoptosis through the NF-κB, PI3K/Akt, and JAK2/STAT3 pathways in laryngeal squamous carcinoma cells [107]. Moreover, histone deacetylase inhibitors valproic acid (VPA) and trichostatin A (TSA) in PANC1 and PaCa44 pancreatic cancer-derived cell lines triggered autophagy through ROS production [108].

Photodynamic therapy (PDT) is a method for the treatment of tumors, based on a photochemical reaction between a photosensitizer (PS) and molecular oxygen. These three apparently harmless components, taken together, result in the formation of ROS [118]. When the PS, after intravenous, intraperitoneal, or topical administration, is exposed to light with a precise wavelength, it changes from a ground (singlet) state to an excited (triplet) state. The excited state can undergo two kinds of reactions; it can react directly with substrates in the cells, such as the membrane or a molecule, transferring an electron or a proton to form radical anion or cation species (type I reactions), whereby these radicals react with oxygen to form oxidizing free radicals and singlet oxygen [119]. Alternatively, excited PS can be restored to the ground state, which then releases energy inducing the conversion of oxygen to the excited state singlet oxygen. Both species produced exert a cytotoxic effect on the cell, as they both interact with lipids, proteins, and nucleic acids. The irradiation of the tumor can selectively activate the photosensitive drug in situ, triggering a photochemical reaction and tumor destruction, via three different mechanisms: (1) PDT can kill the malignant cells directly, through ROS generation; (2) PDT can damage the tumor-associated vasculature, leading to tumor infarction; (3) PDT can activate an inflammatory and immune response against tumor cells [120]. Focusing on the first mechanism, PDT can evoke apoptosis, necrosis, and autophagy-associated cell death pathways. As an example, mitochondria-associated PSs leading to the photodamage of Bcl-2 is a permissive signal for mitochondrial outer membrane permeabilization (MOMP), mediating the release of caspase activators cytochrome c and Smac/DIABLO or proapoptotic molecules such as apoptosis-inducing factor (AIF) [121]. Moreover, other nonapoptotic pathways could be elicited, including the necrosis signaling cascade [122] and autophagy that can have both a cryoprotective and a pro-death role, depending on the PDT doses [123,124]. However, it has been demonstrated that cancer cells exploit their antioxidant activity to neutralize ROS derived for PDT, as an increase in SODs and other antioxidant enzymes has been observed following PDT [125]. Moreover, PDT induces the expression of proteins that are related to signaling pathways such as apoptosis [126] or are responsible for cell survival mechanisms, in order to cope with the oxidative stress and damage. Transcription factors such as Nrf2, activator protein 1 (AP-1), HIF1, and NF-κB are among the factors that are expressed, in addition to those that mediate the proteotoxic stress response [127,128]. New combinatorial approaches to increase the efficacy of the therapy are now being studied, while also integrating chemotherapeutic drugs and PSs into nanocarriers [129,130]. Developed on the basis of PDT, sonodynamic therapy (SDT) is a novel noninvasive approach for use against solid tumors, with low-intensity ultrasound and sonosensitizers [131,132], inducing an excess of ROS, thereby promoting cell death pathways via downregulation of Bcl-2 family proteins [133]. Lastly, new ways to improve traditional PDT are being developed; since PDT has limited killing capacity due to hypoxia in the tumor niche, strategies are taken into consideration not only to increase the ROS killing effect, but also to inhibit ROS defense systems (Figure 2 and Figure 3) [134].

## 7. Conclusions and Future Perspectives

In this review, we discussed the currently known intracellular pathways mediated either by mitochondrial structure rearrangements or by mitochondrial ROS production and release. In particular, we demonstrated how finely tuned the regulation of mitochondrial shaping is, reporting the presence of a two-way modulation of mitochondrial dynamics by several pathways and the existence of a vice versa axis [23,27,28,29,30]. Interestingly, these two players can also establish more intricated positive feedback loops or vicious cycles, directly responsible for the maintenance of physiological states or contributing to pathological conditions. For instance, we reported that mitochondria can restore their own biogenesis in normal tissues through an upregulation of canonical Wnt via the Pgam5/β-catenin interaction, which stimulates mitophagy and organelle remodeling [27], while a mitochondria/Notch cascade alters mitochondrial fusion and fission rates, ultimately supporting tumor proliferation [29].

Moreover, we addressed the possibility of exploring the functional role of “contactology” in cell signaling modulation, especially to unravel possible links with disease formation and development. Nevertheless, we believe that mitochondrial biology is now evolving into “organellar biology”, via which several different organelles work together to regulate important intracellular pathways. In this regard, further studies may be helpful to more deeply investigate the role of ER/mitochondria in cell signaling modulation, but further experiments will be necessary to address this issue.

The existence of a direct link between mitochondrial ROS and cell signaling was also reported in this review, resulting in the modulation of important cellular functions such as proliferation, autophagy, and apoptosis, also acting on a transcriptional level, as summarized in Figure 2 and Figure 3. This leads to the possibility of taking advantage of mitochondrial ROS production for anticancer treatment in multiple ways, by both lowering and enhancing mitochondrial ROS levels inside the cells, resulting in the promotion of cell death via, for instance, DNA damage or mitochondrial impairment, which ultimately provokes the block of tumor progression [68]. To support the idea of the efficacy of this strategy, we collected the novel findings on mitochondrial ROS-targeting drugs (Table 1) which proved to be useful in in vitro studies and could be possibly employed for future clinical trials. The presence of innovative approaches, such as the introduction of photodynamics [118,119,120,121] and sonodynamics [131,132,133], to specifically activate mitochondrial ROS targeting pharmaceuticals to treat cancer supports the relevance of the exploitation of this molecular species, underlining the importance of dissecting cell signaling cascades in which they are involved.

In conclusion, it is clear that mitochondrial physiology has a fundamental role in tuning intracellular functions, leading to the possibility to target these organelles to treat several human diseases. Further work will be necessary to improve drug selectivity to preferentially hit pathological cells while sparing healthy ones.

## Figures and Tables

**Table 1:** Novel pharmaceutical treatments based on mitochondrial ROS exploitation which proved to be effective in cancer management.

Pharmacological Treatments	Cancer Types	Cell Lines	Mechanism of Action	Reference
Resveratrol + salinomycin	Breast cancer	MCF-7	↑ ROS impairs mitochondrial membrane potential; decreased Bcl2 expression, activation of caspases 7,8,9, chromatin condensation, PARP cleavage, apoptosis	[100]
Resveratrol + salinomycin	Breast cancer	MCF-7	↑ ROS activates MAPK pathway, phosphorylates JNK and p38, leading to apoptosis	[100]
Withaferin A *(WA)*	Colorectal cancer	HCT-116, RKO	↑ ROS reduces mitochondrial membrane potential, decreasing Bcl-2/Bax ratio, activating caspase 3–9, leading to apoptosis, and activating JNK pathway	[101]
Carnosic Acid *(CA)*	Colon cancer	HCT-116	↑ ROS diminishes STAT3 phosphorylation, decreasing STAT3 gene products	[76]
Quinalizarin	Breast cancer	MCF-7	↑ ROS affects MAPK, STAT3, and NF-κB signaling pathways, inducing cell-cycle arrest and apoptosis	[102]
Quinalizarin	Lung cancer	A549	↑ ROS affects MAPK, STAT3, and NF-κB signaling pathways, inducing cell-cycle arrest and apoptosis	[103]
Cucurbitacin *(CuD)*	Pancreatic cancer	Capan-1	↑ ROS induces G2/M cell-cycle arrest and mediates p38/MAPK pathway, promoting cell death	[104]
Imiquimod *(IMQ)*	Skin cancer	BCC/KMC-1, B16F10 and A375	↑ ROS causes mitochondrial membrane potential loss, mitochondrial fission, and mitophagy	[105]
Isorhamnetin *(IH)* + chloroquine *(CQ)*	Breast cancer	MDA-MB-231, MCF-7, BT549, MCF-10A	ROS-mediated phosphorylation of CaMKII/Drp1 promotes Bax translocation and release of cytochrome c, mitochondrial fission, caspase activation, and apoptosis	[106]
Cetuximab + oridonin	Laryngeal cancer	Hep-2, Tu212	↑ ROS, through NF-κB, PI3K/Akt, and JAK2/STAT3, induces apoptosis	[107]
Valproic acid *(VPA)* + Trichostatin A *(TSA)*	Pancreatic cancer	PANC1, PaCa44	↑ ROS triggers autophagy	[108]

Legend: ↑: increase; ROS: reactive oxygen species; Bcl-2: B-cell lymphoma 2; PARP: poly adenosine phosphate-ribose polymerase; MAPK: mitogen activated protein kinase; JNK: c-Jun N-terminal kinase; Bcl-2/Bax; B-cell lymphoma 2/Bcl-2-associated X protein; STAT3: signal transducer and activator of transcription; NF-κB: nuclear factor kappa-light-chain-enhancer of activated B cells; CaMKII/Drp1: Ca^2+^/calmodulin-dependent protein kinase II/Dynamin-1-like protein; PI3K/Akt: Phosphatidylinositol 3 Kinase/Protein Kinase B; JAK2: Janus kinase 2.
